# Facile synthesis of boronic acid-functionalized magnetic nanoparticles for efficient dopamine extraction

**DOI:** 10.1186/s40580-019-0200-7

**Published:** 2019-09-02

**Authors:** Jeong Keun Kook, Viet-Duc Phung, Do-Yeong Koh, Sang-Wha Lee

**Affiliations:** 1 Department of Chemical and Biological Engineering, Gachon University, 1342 Seongnamdaero, Sujeong-gu, Seongnam-si, 13120 South Korea; 2 Future Materials and Devices Laboratory, Institute of Fundamental and Applied Sciences, Duy Tan University, 10C, Tran Nhat Duat Street, District 1, Ho Chi Min City, 70000 Vietnam

**Keywords:** Dopamine, Boronic acid, Core–shell, Dopamine, Polydopamine, Fluorescent intensity

## Abstract

Because dopamine (DA) is one of the most critical neurotransmitters that influence a wide variety of motivated human behaviors, it is necessary to develop a facile diagnostic tool that can quantify the physiological level. In this study, core–shell magnetic silica nanoparticles (Fe_3_O_4_@SiO_2_) were prepared using a modified sol–gel reaction. The Fe_3_O_4_@SiO_2_ were functionalized using 3-aminophenylboronic acid (APBA) via a facile and rapid synthetic route, hereafter referred to as Fe_3_O_4_@SiO_2_@APBA The resultant Fe_3_O_4_@SiO_2_@APBA not only adsorbed DA molecules, but also were easily separated from solution using a simple magnetic manipulation. The adsorbed amounts of DA by the Fe_3_O_4_@SiO_2_@APBA were quantified by measuring the changes in fluorescence intensity of polydopamine (at 463 nm) originated from the self-polymerized DA remained in the supernatant before and after the adsorption process. The Fe_3_O_4_@SiO_2_@APBA exhibited two-stage adsorption behavior for DA, and the maximal adsorption capacity was 108.46 μg/g at pH 8.5. Our particle system demonstrated the potential application for extracting compounds with cis-diols (including catechol amines) from the biological fluid.

**Electronic supplementary material:**

The online version of this article (10.1186/s40580-019-0200-7) contains supplementary material, which is available to authorized users.

## Introduction

Dopamine (DA) is one of the most important catecholamine neurotransmitters, which plays major roles in central nervous system processes, such as rewarding-motivated behavior in the brain, regulating exercise, and controlling the release of various hormones [1–4]. Since the DA system plays a key role in several medical conditions, such as Parkinson’s disease, Segawa disease, schizophrenia, and attention deficit hyperactivity disorder, DA concentration in biological fluids is used as important indicator for early disease diagnosis [5, 6]. Several analytical methods have been used to detect DA molecules in bio-fluids, including high-performance liquid chromatography (HPLC), enzyme-linked immune sorbent assay, fluorescence measurements, electrochemical analysis, and surface-enhanced Raman scattering (SERS) [7–12]. However, it is very difficult to determine physiological levels of DA owing to its low concentration and the presence of interfering components, which can decrease the effectiveness of common analytical tools [13, 14]. Therefore, it is essential to separate target molecules from co-existing interfering components for accurate analysis.

Currently, solid-phase extraction (SPE) is the preferred method for concentrating and purifying small amounts of analytes from biological fluids [15, 16]. In particular, magnetic solid-phase extraction (MSPE) is a new process for pre-concentrating target analytes using magnetic adsorbents [17]. The MSPE process has been applied in a wide range of fields for the separation of various materials because the adsorbent material does not need to be packed into any type of device, unlike traditional SPE [18, 19]. Therefore, there has been a great interest for developing adsorbent materials consisting of a magnetite core and silica (or polymer) shell. In particular, the silica surface is easily activated and can anchor various functional groups because of its abundant Si–OH groups [19, 20]. Boronic acid is also known as a typical adsorbent for catecholamine extraction because it can form cyclic esters with cis-diol compounds under alkaline conditions [21–24]. Thus, boronic acid-functionalized materials exhibit strong affinity toward cis-diol-containing compounds, which leads to their applications for various functional materials and tasks, such as diagnostic sensing, magnetic separation, and targeted drug delivery [25, 26]. When preparing boronic acid-functionalized materials, however, amine-terminated matrices should be conjugated with 4-formylphenylboronic acid via reductive amination, which typically requires very long reaction times (more than 3 days) [27–30].

In this study, core–shell magnetic silica nanoparticles (Fe_3_O_4_@SiO_2_) featuring surface amine groups were facilely prepared via sol–gel reactions, and the amine-terminated Fe_3_O_4_@SiO_2_ (Fe_3_O_4_@SiO_2_–NH_2_) were subsequently modified into carboxylic acid-terminated Fe_3_O_4_@SiO_2_ (Fe_3_O_4_@SiO_2_–COOH) via a linker elongation reaction with succinic anhydride (SA). Furthermore, the Fe_3_O_4_@SiO_2_–COOH was conjugated with 3-aminophenylboronic acid (APBA) for 5 h. Boronic acid-functionalized Fe_3_O_4_@SiO_2_ (Fe_3_O_4_@SiO_2_@APBA) were not only magnetically separable, but also form cyclic esters with the cis-diol groups of DA. The Fe_3_O_4_@SiO_2_@APBA exhibited two-stage adsorption behavior, and the maximal adsorption capacity of DA was 108.46 μg/g at pH 8.5.

## Experimental methods

### Chemicals

All solvents were of analytical grade and were used as received. Iron(III) chloride hexahydrate (FeCl_3_·6H_2_O), iron(II) chloride tetrahydrate (FeCl_2_·4H_2_O), sodium citrate tribasic dehydrate, 3-aminopropyl trimethoxysilane (APTMS, 97%), tetraethyl orthosilicate (TEOS), ammonia hydroxide (NH_4_OH, 29 wt%), APBA, SA, N,N-dimethyl formamide (DMF), N-(3-dimethylaminopropyl)-Nʹ-ethylcarbodiimide hydrochloride (EDC), N-hydroxysulfosuccinimide sodium salt (NHS) and tris–HCl buffer (pH 8.0) were purchased from Sigma-Aldrich. Deionized (DI) water (HPLC grade) was obtained from Daejung Co.

### Amine-terminated magnetic nanoparticles (Fe_3_O_4_@SiO_2_–NH_2_)

The core–shell magnetic silica nanoparticles (Fe_3_O_4_@SiO_2_) were synthesized by first mixing FeCl_3_·6H_2_O and FeCl_2_·4H_2_O (3:2 mol ratio) with DI water. Next, 20 mL NH_4_OH (29 wt%) was added quickly to the iron chloride mixture under vigorous stirring at room temperature (RT), and black magnetite precipitates were formed immediately after the addition of NH_4_OH. The precipitates were then mixed with citrate solution (1.0 M) for 3 h at RT, and the solution was refluxed for 2 h at 70 °C to obtain citrate-capped magnetite (C-Fe_3_O_4_) [31–34]. Afterward, APTMS-complexed magnetite (A-Fe_3_O_4_) particles were prepared by adding APTMS to the C-Fe_3_O_4_ solution at RT, which formed magnetite clusters featuring terminal alkoxy groups [35]. Then, 1.0 mL A-Fe_3_O_4_ was added to 80 mL ethanol that contained 5 mL NH_4_OH (29 wt%), followed by the rapid injection of 0.2 mL TEOS under vigorous stirring for 18 h at 30 °C. Next, 0.2 mL APTMS was added to the mixture under vigorous stirring for 2 h at 30 °C, followed by refluxing for 2 h at 70 °C to strongly anchor the surface amine groups. After the reaction product was centrifuged three times using ethanol and was subsequently dispersed in 30 mL ethanol, the Fe_3_O_4_@SiO_2_–NH_2_ were obtained.

### Boronic acid-functionalized Fe_3_O_4_@SiO_2_ (Fe_3_O_4_@SiO_2_@APBA)

First, 500 μL Fe_3_O_4_@SiO_2_–NH_2_ was added dropwise to 30 mL DMF that contained 10% SA and the mixture was stirred continuously for 5 h by continuously purging N_2_ gas through it [36–38]. Afterward, the obtained Fe_3_O_4_@SiO_2_–COOH were washed with acetone several times. The purified Fe_3_O_4_@SiO_2_–COOH were dispersed in 30 mL DI water. Then, 30 mL Fe_3_O_4_@SiO_2_–COOH dispersion was mixed with 24 mg EDC and 27 mg NHS, followed by the addition of 8 mg APBA under continuous stirring for 5 h [39, 40]. After the reaction product was centrifuged and washed with DI three times, Fe_3_O_4_@SiO_2_@APBA were finally obtained. The product sample was subsequently dispersed in tris–HCl buffer prior to further characterization.

### Measurement of DA concentration

The concentration of DA was determined by measuring the changes in fluorescence intensity of polydopamine (PDA) [41, 42]. Under alkaline pH condition, DA is transformed into the oxidized form of quinone, which self-polymerizes into polydopamine (PDA). Fluorescence spectra of PDA were recorded in the visible wavelength ranged of 400–600 nm at the excitation wavelength of 370 nm. Then, the DA concentration was determined by measuring the fluorescence intensity of PDA at 463 nm, using photoluminescence spectroscopy, based on the linearly regressed standard curve between the fluorescence intensity of PDA and the corresponding DA concentration [43].

### Instrumental analysis

The sizes and morphologies of the samples were analyzed using a scanning electron microscopy (SEM, 15 kV, S-4700; Hitachi, Japan) instrument and transmission electron microscopy (TEM, 200 kV, H-7600; Hitachi, Japan) device, and the magnetic properties of the samples were measured using a vibrating sample magnetometer (VSM, model 7404; Lake Shore, USA). The surface functional groups and zeta potentials of the particles were analyzed using a Fourier-transform infrared (FTIR) spectroscopy (Vertex 70; Bruker, Germany) and zeta potential meter (Otsuka Electronics, Japan), respectively.

## Results and discussion

**Scheme 1:**
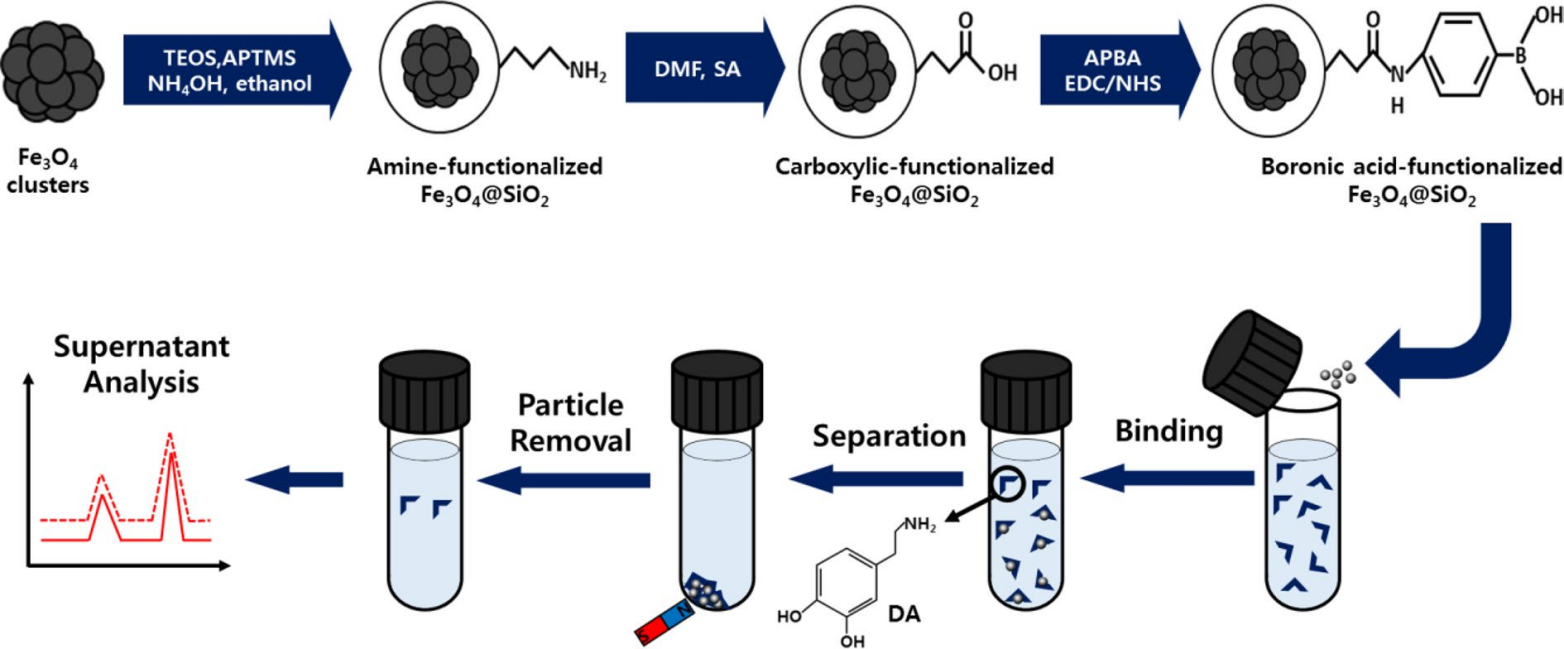
Synthetic scheme of boronic acid-functionalized magnetic silica (Fe_3_O_4_@SiO_2_@APBA) nanoparticles, simple magnetic separation, and fluorescence detection of dopamine in supernatant. Here, TEOS, APTMS, DMF, SA, APBA, EDC, and NHS are tetraethyl orthosilicate, aminopropyl trimethoxysilane, N,N-dimethyl formamide, succinic anhydride, 3-aminophenylboronic acid, N-(3-dimethylaminopropyl)-Nʹ-ethylcarbodiimide hydrochloride, and N-hydroxysulfosuccinimide sodium salt, respectively

Scheme 1 depicts the synthetic procedure of Fe_3_O_4_@SiO_2_@APBA, adsorption of DA on Fe_3_O_4_@SiO_2_@APBA, and final detection of residual DA in the supernatant. First, C-Fe_3_O_4_ was complexed with APTMS ligands to form A-Fe_3_O_4_. Additional file 1: Fig. S1 presents the clustered morphology of A-Fe_3_O_4_. Subsequently, A-Fe_3_O_4_ was coated with a silica layer via a sol–gel reaction with TEOS to obtain the Fe_3_O_4_@SiO_2_, followed by functionalization with surface amine groups. The Fe_3_O_4_@SiO_2_–NH_2_ was converted into the Fe_3_O_4_@SiO_2_–COOH via a linker elongation reaction with SA. Lastly, APBA was conjugated with the Fe_3_O_4_@SiO_2_–COOH and formed the Fe_3_O_4_@SiO_2_@APBA. Then, the Fe_3_O_4_@SiO_2_@APBA was used to extract DA in tris–HCl buffer solution (at pH 8.5). Afterward,, the supernatant was separated from the Fe_3_O_4_@SiO_2_@APBA using an external magnet. The residual DA in the supernatant was quantified based on the fluorescence intensity of PDA, which formed from the self-polymerized DA under alkaline conditions. Our chemical strategy combined a facile and rapid synthetic route with simple fluorescence analysis, which could compensate the drawbacks of conventional methods that typically require long preparation times and complex analysis procedures for trace DA detection.

In the fabrication of core–shell Fe_3_O_4_@SiO_2_, more addition of silica precursor relative to Fe_3_O_4_ leads to the size increase of particles with more uniformity and lower magnetism. On the other hand, less addition of silica precursor leads to the size decrease of particles with less uniformity and larger magnetism, which can make it more difficult to control the binding process between DA and the particles. Thus, a certain size of particles with an appropriate magnetism needs to be prepared to enhance the binding process and produce uniform particle size.

**Fig. 1:**
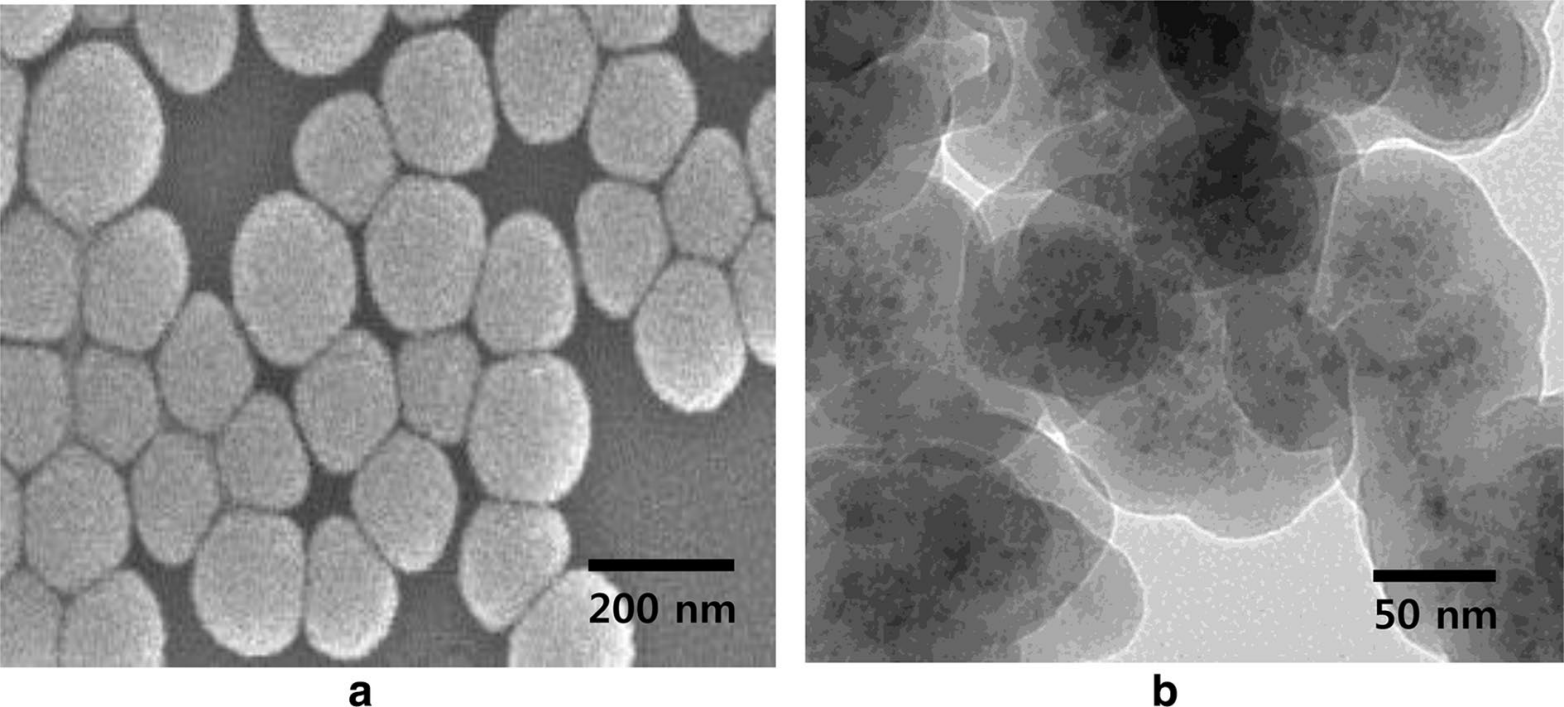
**a** Scanning electron microscopy image of Fe_3_O_4_@SiO_2_ and **b** transmission electron microscopy images of Fe_3_O_4_@SiO_2_

The size and morphology of the core–shell Fe_3_O_4_@SiO_2_ were studied using SEM and TEM, respectively. According to the SEM image in Fig. 1a, the Fe_3_O_4_@SiO_2_ exhibited uniform and spherical shapes. The average particle size was estimated to be 190 ± 9 nm using dynamic light scattering (Additional file 1: Fig. S2). The TEM image of Fe_3_O_4_@SiO_2_ illustrated the distinct embedding of Fe_3_O_4_ clusters into the silica matrix (Fig. 1b). The thickness of the silica shell was estimated to be ~ 30 nm by analyzing the TEM image.

**Fig. 2:**
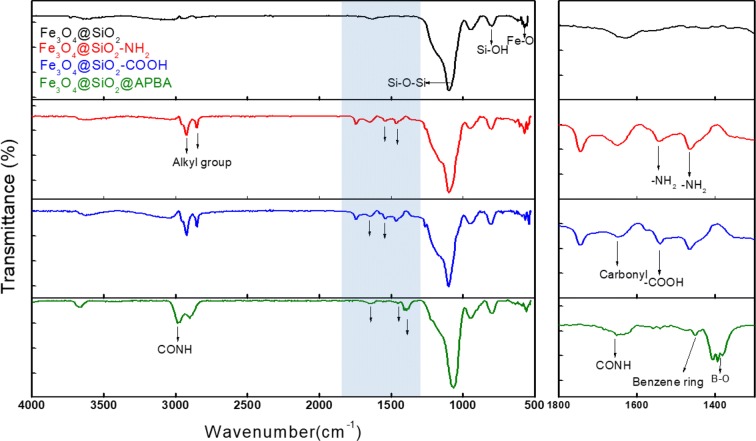
Fourier-transform infrared spectra of as-prepared samples

Figure 2 illustrates the FTIR spectra of the as-prepared samples for each step of the synthesis process. The Fe_3_O_4_@SiO_2_ presented FTIR bands that corresponded to the stretching vibrations of Si–O–Si (1085 cm^−1^) and Fe–O (570 cm^−1^). On the other hand, Fe_3_O_4_@SiO_2_–NH_2_ exhibited new peaks at 1550 and 1450 cm^−1^, which were attributed to the amine groups of APTMS. In addition, the peaks at 2923 and 2851 cm^−1^ were assigned to the asymmetric and symmetric C–H stretching vibrations of aliphatic –CH_2−_, respectively. Moreover, Fe_3_O_4_@SiO_2_–COOH, which formed during the coupling reaction of Fe_3_O_4_@SiO_2_–NH_2_ with SA in DMF, exhibited additional peaks at 1640 and 1600 cm^−1^, which corresponded to the carbonyl and carboxylic acid groups of conjugated SA, respectively. The zeta potentials, ζ, of the samples presented alternating changes in surface charge depending on the surface functional groups, which indicated the successful modification of the particles: the ζ values of Fe_3_O_4_@SiO_2_, Fe_3_O_4_@SiO_2_–NH_2_, and Fe_3_O_4_@SiO_2_–COOH were − 18.78 ± 2.13, 14.25 ± 2.27, and − 41.38 ± 2.08, respectively (see Additional file 1: Fig. S3). In addition, Fe_3_O_4_@SiO_2_@APBA exhibited peaks at 1650 and 3000 cm^−1^, which were ascribed to the amide groups (CONH) and indicated the successful coupling reaction between the carboxyl groups of Fe_3_O_4_@SiO_2_-COOH and amine groups of APBA. Furthermore, Fe_3_O_4_@SiO_2_@APBA also exhibited peaks at 1380 cm^−1^ (B–O stretching) and 1450 cm^−1^ (benzene ring), which indicated the presence of boronic acid functional groups, as illustrated in the magnified images in the right column of Fig. 2.

**Fig. 3:**
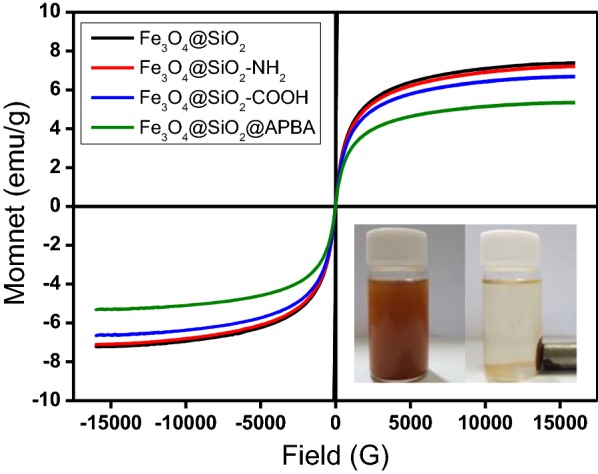
Hysteresis loops of as-prepared samples featuring different surface functional groups

The saturated magnetization curves of the samples were obtained using a VSM. As presented in Fig. 3, the functionalized particles exhibited lower magnetization values than the pristine Fe_3_O_4_@SiO_2_ and the magnetization values of the samples decreased as follows: Fe_3_O_4_@SiO_2_ (7.2 emu/g) > Fe_3_O_4_@SiO_2_–NH_2_ (7.1 emu/g) > Fe_3_O_4_@SiO_2_–COOH (6.6 emu/g) > Fe_3_O_4_@SiO_2_@APBA (5.3 emu/g). All samples exhibited superparamagnetic characteristics of negligible remanence and coercivity, i.e., no hysteresis was observed in the magnetization curves. The saturated magnetisms of as-prepared particles were decreased with the increase of surface modification steps, due to the addition of nonmagnetic organic materials. After the Fe_3_O_4_@SiO_2_@APBA were dispersed in solution, an external magnet was placed near the bottle containing the particles. As expected, the particles were attracted toward a spot near the external magnet. The complete separation of the Fe_3_O_4_@SiO_2_@APBA using the external magnet was achieved within 2 min. The pictorial demonstration of the magnetic separation of the Fe_3_O_4_@SiO_2_@APBA is illustrated in the inset of Fig. 3.

The XRD patterns of A-Fe_3_O_4_ and Fe_3_O_4_@SiO_2_ are shown in Additional file 1: Figure S4. All the XRD peaks of A-Fe_3_O_4_ are corresponding to (220), (311), (400), (422), (511) and (440) planes of Fe_3_O_4_ phase (JCPSD Card No. 76-0956). In contrast, Fe_3_O_4_@SiO_2_ exhibited the broad peak at ~ 22.5° that revealed the distinct amorphous silica phase, but did not show the distinct XRD peaks of Fe_3_O_4_ phase, indicative of the successful synthesis of core–shell nanostructure consisting of silica layer and clustered Fe_3_O_4_ core.

The colloidal stability of Fe_3_O_4_@SiO_2_@APBA is important for their adsorption capacity for DA. The Fe_3_O_4_@SiO_2_@APBA were dispersed in five different media (DI water, phosphate buffered saline (PBS) (pH 7.4), tris–HCl buffer (pH 8), tris–HCl buffer (pH 9), and ethanol) at RT. The dispersion degree of the particles was monitored after maintaining them in each media for 3 h, and then the mixtures were re-dispersed to check for the formation of precipitates in each bottle. According to the pictorial diagrams presented in Additional file 1: Fig. S5(a), (b), and (c), the dispersion degree of the particles increased as follows: ethanol < DI water < PBS (pH 7.4) ≅ tris–HCl (pH 9) < tris–HCl (pH 8). The lowest degree of particle dispersion was obtained for the ethanol mixture, whereas the highest degree of particle dispersion was obtained for the tris–HCl (pH 8.0) mixture. For the tris–HCl (pH 9.0) and PBS (pH 7.4) dispersions, some aggregation was observed at the bottom of the bottle after repeated dispersion/re-dispersion steps. Because the pKa value of APBA is ~ 8.6, the decrease in particle dispersion in the tris–HCl buffer (pH 9.0) mixture was probably due to the higher surface reactivity of APBA at pH 9.0 than that at pH 8.0.

**Fig. 4:**
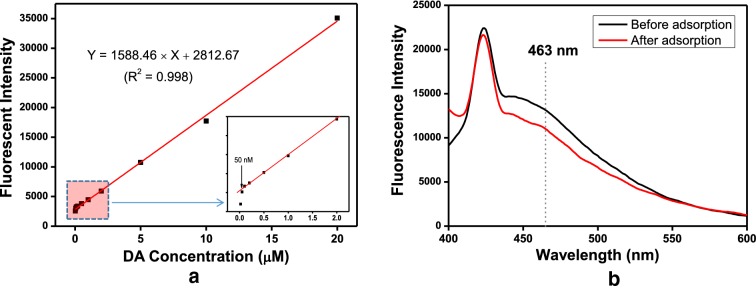
**a** Standard curve of dopamine (DA) concentration vs. fluorescence intensity of polydopamine (PDA) at 463 nm. **b** Comparative fluorescence spectra of PDA obtained from DA that remained in supernatant before and after adsorption using 3-aminophenylboronic acid-functionalized magnetic silica (Fe_3_O_4_@SiO_2_@APBA) nanoparticles at pH 8.5

Figure 4a illustrates the fluorescence intensity of converted PDA at 463 nm as a function of DA concentration. The standard curve was calibrated over the concentration range of 0.02–20 μM, but the standard curve deviated from linearity at the lowest concentration, below 50 nM, according to the inset of Fig. 4a. Afterwards, the adsorption process was carried out by adding 4 mg of Fe_3_O_4_@SiO_2_@APBA to 2 mL DA solution. The supernatant contained the residual DA that was not adsorbed by the Fe_3_O_4_@SiO_2_@APBA.

DA is converted into the oxidized form of quinone product in the alkaline condition (at ~ pH 12), which is rapidly polymerized to polydopamine (PDA). Thus, the fluorescent intensity of PDA at 463 nm can be quantitatively related to the DA concentration of the sample using the linearly regressed standard curve between the fluorescent intensity of PDA, Y, and the corresponding DA concentration, X: X = (Y-2812.67)/1588.46 (R^2^ = 0.998), where the unit of X is μg of DA/mg of particles. Finally, the adsorption capacity of Fe_3_O_4_@SiO_2_@APBA was calculated by converting the difference of fluorescent intensity before and after the adsorption of DA by the Fe_3_O_4_@SiO_2_@APBA, as shown in Fig. 4b.

**Fig. 5:**
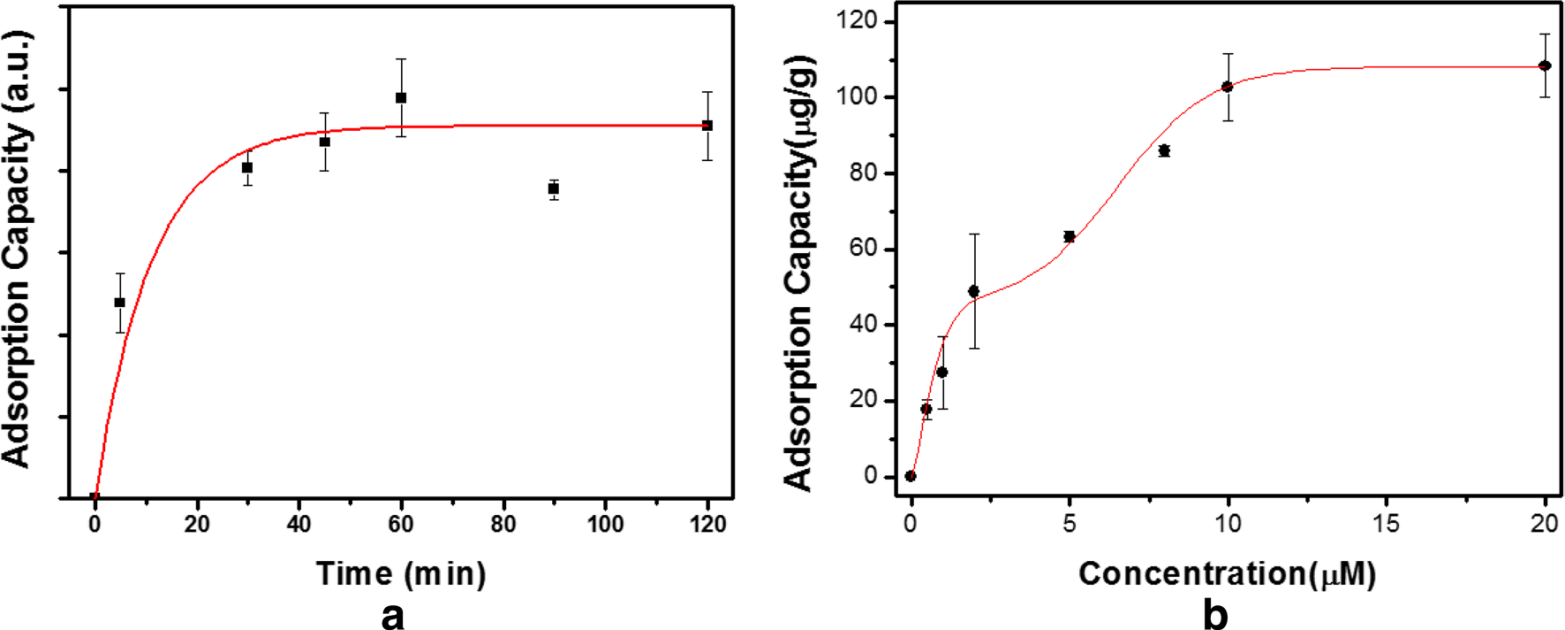
**a** Adsorption kinetics of dopamine using 3-aminophenylboronic acid-functionalized magnetic silica (Fe_3_O_4_@SiO_2_@APBA) at pH 8.5. **b** Adsorption isotherms of Fe_3_O_4_@SiO_2_@APBA for dopamine at pH 8.5

Additional file 1: Fig. S6 illustrates the effect of pH on the adsorption capacity of Fe_3_O_4_@SiO_2_@APBA for DA (2.0 μM) in tris–HCl buffer solution (pH 7.0–9.5). Even though the adsorption capacity of Fe_3_O_4_@SiO_2_@APBA turned out be maximal at pH 9.0, the adsorption process was carried out at pH 8.5 owing to the sensitive reactivity of DA under strong alkaline conditions (pH 9.0–9.5). According to the data in Fig. 5a, the adsorption capacity increased during the first 30 min and gradually reached saturation within 60 min. Thus, the equilibrium capacity of DA was achieved after 60 min of adsorption time. During the adsorption process, the borate groups were transformed into tetrahedral anionic forms which could form cyclic esters with DA molecules.

To evaluate their adsorption performance, the Fe_3_O_4_@SiO_2_@APBA (4 mg) were added to 2 mL tris–HCl buffer solution (pH 8.5) containing different DA concentrations (0.5–20 nmol/mL). The mixed solution was gently shaken for 60 min and the supernatant was separated from the magnetic particles using an external magnetic field. Then, the adsorption capacity was calculated based on the changes in the fluorescence intensity of PDA before and after the adsorption process. As depicted in Fig. 5b, the Fe_3_O_4_@SiO_2_@APBA exhibited two stage adsorption behavior for DA, and presented a distinct inflection point at ~ 5 μM, which was followed by the gradual increase to the saturated adsorption capacity value of 108.46 μg/g [27, 44–46].

According to Additional file 1: Fig. S7(a), the chemical state of DA changed depending on the solution pH [47]. At pH 8.5, the chemical structure of DA is H_3_DA^−^ and H_2_DA. Thus, the exposed amine groups of DA anchored onto the Fe_3_O_4_@SiO_2_@APBA could interact with the negative hydroxyl groups of the free DA in solution, which consequently lead to the additional adsorption of DA (see Additional file 1: Fig. S7(b)). It is plausible to assume that the adsorption capacity of Fe_3_O_4_@SiO_2_@APBA is attributed to the monolayer adsorption of DA via cyclic ester formation and additional adsorption via electrostatic interactions between DA molecules.

## Conclusions

In this study, the Fe_3_O_4_@SiO_2_@APBA were facilely prepared for the efficient adsorption of DA molecules. First, core–shell Fe_3_O_4_@SiO_2_ were synthesized according to the modified sol–gel reaction between C-Fe_3_O_4_ and TEOS, and the surface of Fe_3_O_4_@SiO_2_ was functionalized using APTMS with primary amine groups. The Fe_3_O_4_@SiO_2_–NH_2_ was further modified into the Fe_3_O_4_@SiO_2_–COOH via a linker elongation reaction with succinic amide. The boronic acid-functionalized Fe_3_O_4_@SiO_2_ were finally prepared by conjugating APBA with the Fe_3_O_4_@SiO_2_–COOH using a EDC/NHS coupling agent. The Fe_3_O_4_@SiO_2_@APBA exhibited two-stage adsorption behavior for DA, and the maximal adsorption capacity was 108.46 μg/g at pH 8.5. Moreover, the Fe_3_O_4_@SiO_2_@APBA was rapidly separated using a simple magnetic manipulation process. Our particle systems demonstrated high affinity for DA molecules, which would make them suitable for extracting compounds (including catecholamine neurotransmitters) with cis-diols from the biological fluid.

## Additional file

**Additional file 1.** Additional figures.

## Data Availability

Not applicable.
