# Supplementary material for: Facile synthesis of boronic acid-functionalized magnetic nanoparticles for efficient dopamine extraction
Source: Nano Converg. 2019 Sep 2;6:30. doi: 10.1186/s40580-019-0200-7 (PMC6717740; doi:10.1186/s40580-019-0200-7)
Supplement: Supplementary file 1 — Additional file 1. Additional figures. [file 40580_2019_200_MOESM1_ESM.docx]

**Facile synthesis of boronic acid-functionalized core-shell magnetic nanoparticles for efficient dopamine extraction**

Jeong Keun Kook, Do-Yeong Koh, Phung Viet Duc, Sang-Wha Lee*

*Department of Chemical and Biological Engineering, Gachon University, 1342 Seongnamdaero, Sujeong-gu, Seongnam-si, 461-701, Republic of Korea*

*E-mail: lswha@gachon.ac.kr, TEL: +82-31-750-5360


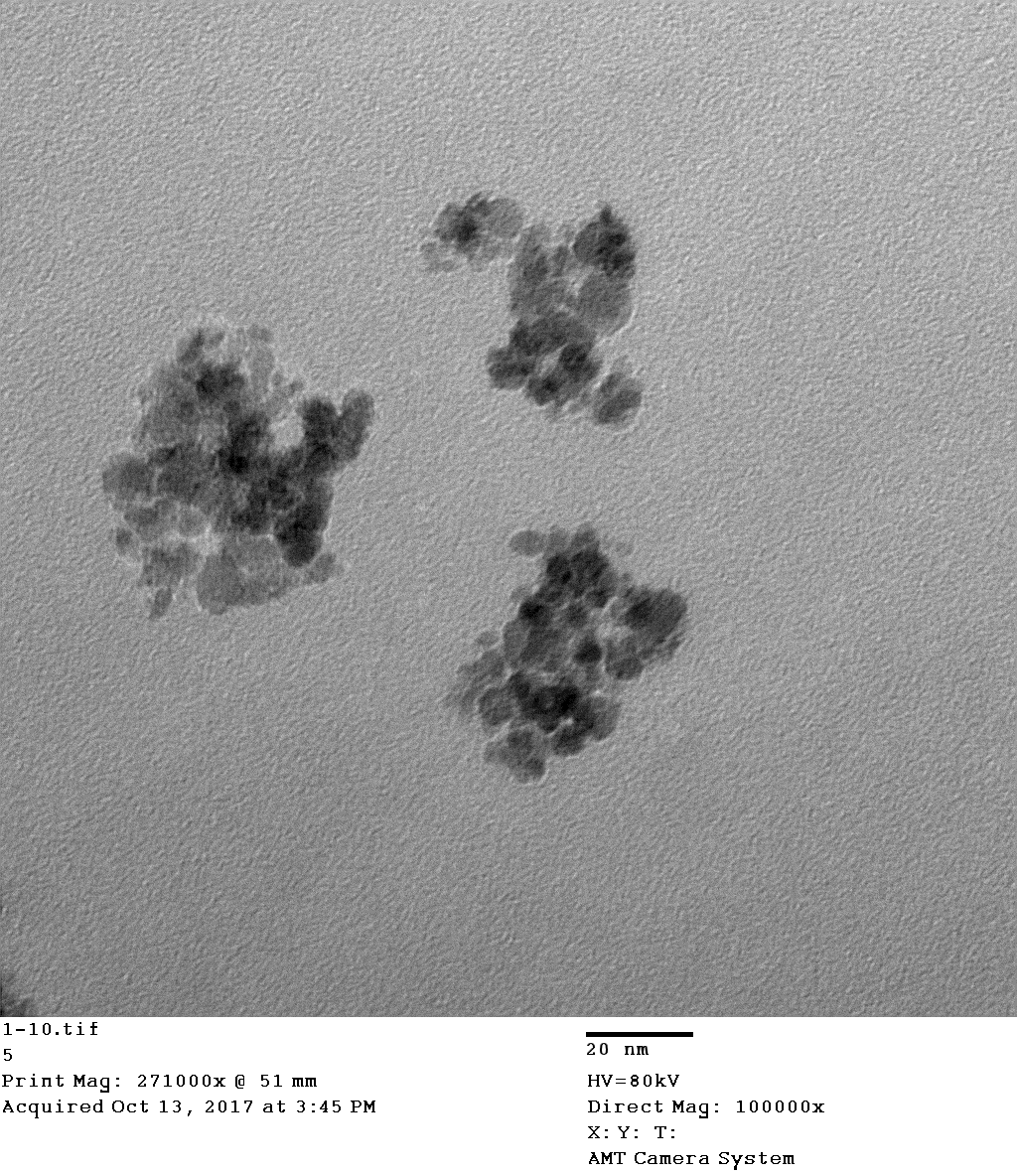


**50 nm**

**Fig. S1.** TEM image of A-Fe_3_O_4_.

**Fig. S2.** Particle size distribution of core-shell Fe_3_O_4_@SiO_2_ by DLS method.


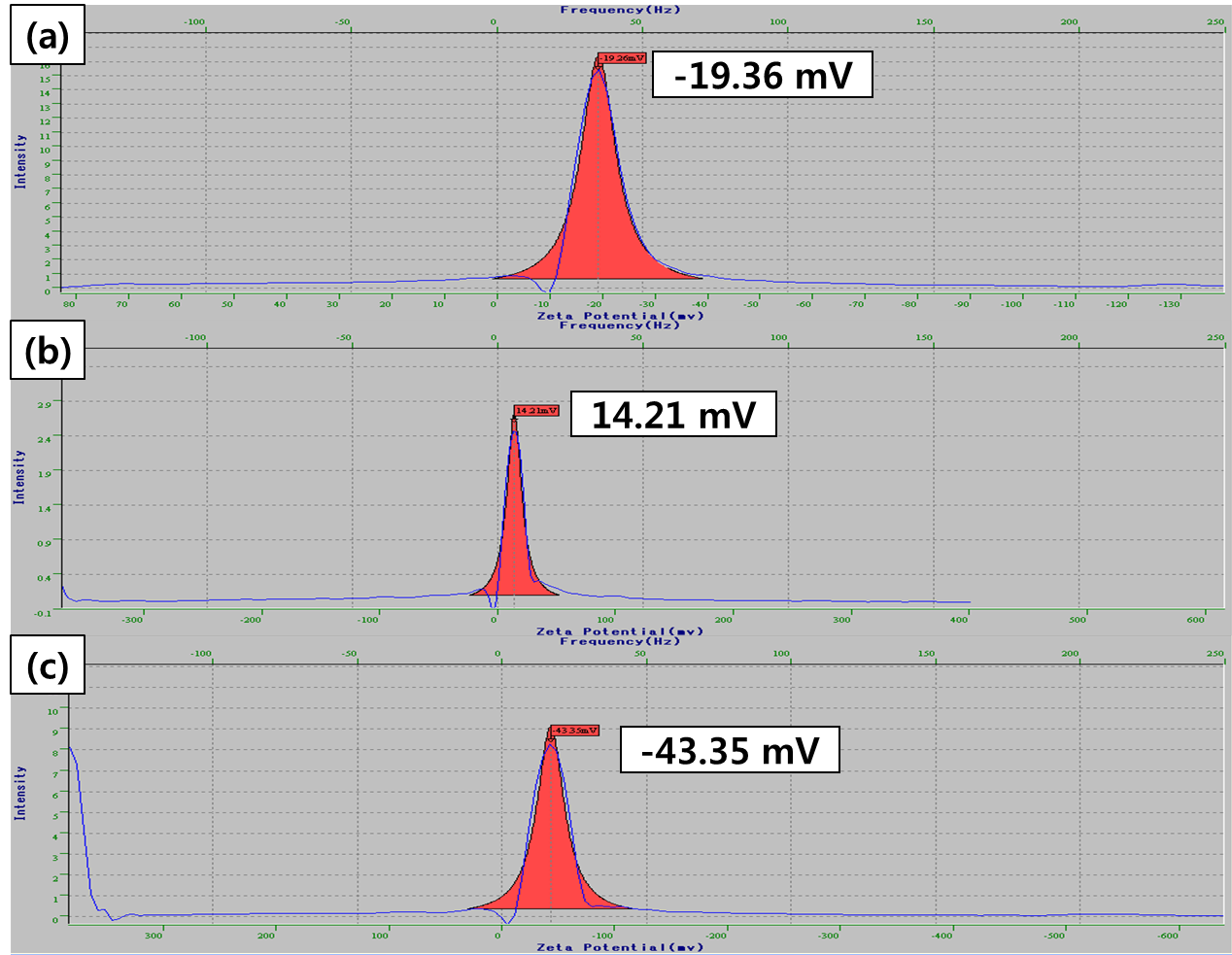


**Fig. S3.** Zeta-potentials of as-prepared samples: a) Fe_3_O_4_@SiO_2_, b) Fe_3_O_4_@SiO_2_–NH_2_ c) Fe_3_O_4_@SiO_2_-COOH.

**Fig. S4.** X-ray diffraction (XRD) patterns of A-Fe_3_O_4_ and Fe_3_O_4_@SiO_2_.

(a) (b) (c)

**Fig. S5.** Comparative dispersion/redispersion of Fe_3_O_4_@SiO_2_@APBA in various medias (**1.** Water, **2.** PBS (pH7.4), **3.** Tris-HCl buffer (pH8.0), **4.** Tris-HCl buffer (pH 9.0), **5.** Ethanol) at different times: a) 0 min, b) 3 hr, c) after re-dispersion .

**Fig. S6.** pH effect on the adsorption capacity of dopamine by Fe_3_O_4_@SiO_2_@APBA.


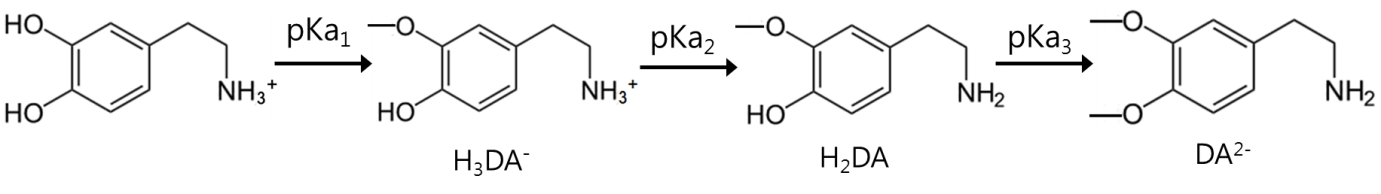


(a)


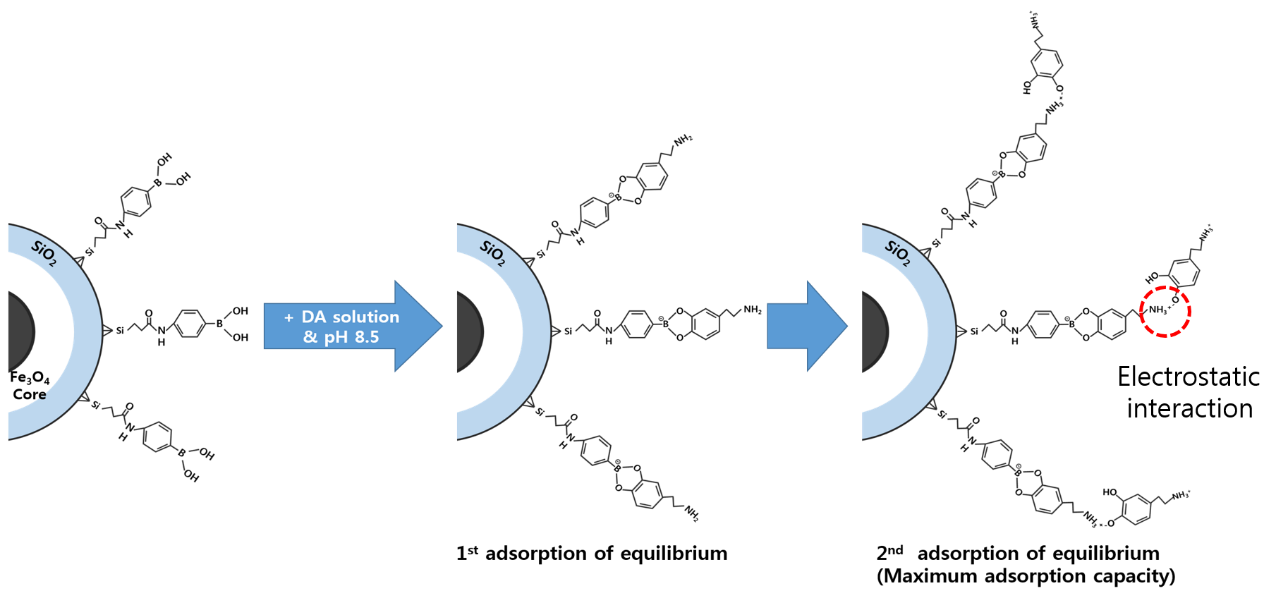


(b)

**Fig. S7.** Adsorption mechanism of dopamine by using boronic aicd-functionalized magnetic particles (Fe_3_O_4_@SiO_2_@APBA).
